# Quantitative evaluation of a deep learning‐based noise reduction algorithm in digital radiography using noise power spectrum analysis

**DOI:** 10.1002/acm2.70521

**Published:** 2026-02-25

**Authors:** Sho Maruyama, Hiroki Saitou

**Affiliations:** ^1^ Department of Radiological Technology Gunma Prefectural College of Health Sciences Maebashi Gunma Japan; ^2^ Department of Medical Radiology Faculty of Medical Technology Teikyo University Itabashi‐ku Tokyo Japan

**Keywords:** deep learning‐based noise reduction, digital radiography, dose‐dependent performance, frequency‐dependent assessment, noise power spectrum

## Abstract

**Background:**

Recently, deep learning (DL)‐based noise reduction (DLNR) has been introduced in clinically used digital radiography (DR) systems, reporting superior performance over conventional algorithms. However, DLNR algorithms often operate as “black boxes” with nonlinear behavior, making it essential to understand the impact of such processing on image quality under different imaging conditions.

**Purpose:**

This study aimed to quantitatively evaluate the image quality of a commercial DLNR algorithm for DR referred to as intelligent noise reduction (INR). Specifically, we compared its noise reduction performance with that of a conventional rule‐based algorithm (conventional noise reduction, Con‐NR) using frequency‐domain metrics with detailed noise power spectrum (NPS) analysis.

**Methods:**

The NPS was used to assess the spatial‐frequency‐dependent behavior of both INR and Con‐NR across varying dose levels and different objects. In this work, we introduced a supplementary metric—the NPS improvement factor (NPSIF)—to quantify noise suppression across frequency ranges and facilitate direct comparison between methods.

**Results:**

The DL‐based algorithm achieved substantial noise reduction at low‐dose settings compared with the conventional method, although its advantages were less pronounced at higher dose levels. The NPSIF effectively captured frequency‐specific differences, thereby offering insights into the strengths and limitations of each technique.

**Conclusions:**

The dose‐dependent performance of the DL‐based algorithm suggests sensitivity to the characteristics of the training data used to develop the DL model. The findings demonstrate distinct differences in the noise suppression behavior between DL‐based and conventional methods in DR and underscore the importance of detailed frequency‐domain evaluation for understanding advanced image processing. Further research is warranted to integrate noise analysis with diagnostic performance metrics to comprehensively assess clinical utility.

## 1 INTRODUCTION

Digital radiography (DR) is extensively used in clinical practice. Although the patient radiation dose per examination in DR is lower than that of other modalities, such as computed tomography (CT), DR contributes to cumulative radiation dose owing to its high accessibility.^1^, ^2^, ^3^ Therefore, optimization of imaging protocols with respect to the radiation dose in DR remains an important task for medical imaging teams, including radiological technologists and medical physicists.^4^, ^5^, ^6^ In this context, objective assessment of the image quality is crucial role for balancing radiation dose and diagnostic performance.^6^, ^7^

In DR using a flat panel detector (FPD), image noise primarily originates from three components: quantum noise associated with photon statistics, electronic noise from the readout system, and structural noise caused by detector inhomogeneities or fixed patterns.^8^, ^9^ Image noise not only significantly affects the accuracy of diagnostic imaging but also plays an important role in dose optimization.^10^ Therefore, in addition to the development of advanced detector technologies, various noise reduction techniques have been adopted in clinical practice.^11^, ^12^, ^13^

The integration of deep learning (DL) technology into radiology has shown promise in enhancing image quality and enabling radiation dose reduction across multiple imaging modalities.^14^, ^15^, ^16^, ^17^ In DR, DL‐based noise reduction (DLNR) methods have been introduced and are reported to outperform conventional rule‐based noise reduction algorithms in improving image quality.^18^, ^19^ Recent studies have further evaluated commercial DLNR tools for chest radiography, demonstrating potential dose reduction while maintaining the diagnostic image quality in both phantom and clinical settings.^20^, ^21^, ^22^ Compared to conventional rule‐based image processing methods, the internal mechanisms of DLNR algorithms function as “black boxes,” making it difficult to predict algorithm outputs under varying input conditions.^23^ Therefore, a thorough understanding of how DLNR algorithms alter image noise characteristics is crucial for medical imaging teams. Handling the nonlinear behavior and potential limitations of DLNR methods would not only facilitate safe and reliable clinical implementation but also inform strategies for optimizing patient dose without compromising diagnostic quality.

In this study, we evaluate the image quality of a newly commercialized DLNR algorithm for DR imaging by comparing it with an extensively used conventional processing method. Specifically, we assess the noise reduction performance of the DLNR algorithm across varying dose levels and object conditions using a detailed analysis based on the noise power spectrum (NPS). The NPS is widely used as a comprehensive descriptor of image noise, as it characterizes not only the magnitude but also the frequency distribution of the noise components in the image. Furthermore, this study introduces a new analytical index that utilized this NPS information.

## 2 METHODS

### 2.1 Definition of the NPS improvement factor

In this work, we introduced the NPS improvement factor (NPSIF) metric to quantitatively evaluate the effectiveness of noise‐reduction techniques in medical imaging. This enables the detailed frequency‐domain evaluation of noise characteristics and potential image degradation. The NPSIF is defined as the ratio of the NPS before and after image processing as follows:

(1) $$NPSIF\;\left(u\right)=\frac{NPS_{-}\left(u\right)}{NPS_{+}\left(u\right)}\;,$$

where u denotes the spatial frequency. Here $NPS_{-}\left(u\right)$ represents the NPS calculated from raw detector output images (without processing) and $NPS_{+}\left(u\right)$ denotes the NPS calculated from images obtained by applying the noise reduction algorithm to the same raw data (with processing applied).

The concept of the NPSIF is inspired by the signal‐to‐noise ratio improvement factor (SIF), which has been proposed as a frequency‐dependent indicator to evaluate changes in imaging performance.^24^, ^25^ The NPSIF is explicitly used to characterize noise‐related effects of image processing in this study. An NPSIF value >1 indicates noise reduction at the corresponding spatial frequency, whereas a value <1 indicates increased noise. This metric represents only the influence of noise characteristics on the image signal‐to‐noise ratio (SNR); it does not evaluate other aspects of image quality, such as the spatial resolution.

### 2.2 Calculation of the noise characteristics

**FIGURE 1:**
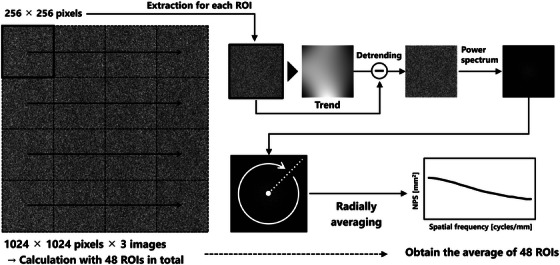
Overview of the procedure of NPS measurement.

NPS measurements for the calculation of the NPSIF were performed in accordance with IEC 62220‐1‐1^26^ using the two‐dimensional (2D) Fourier transform method. An overview of the procedure is shown in Figure 1. A square region of interest (ROI) of 1024 × 1024 pixels was positioned at an area of interest for noise analysis of each image and subdivided into local ROIs of 256 × 256 pixels for individual NPS calculations.

For each local ROI, a 2D trend component was estimated and subtracted from the original pixel values to isolate the fluctuation component. This detrended fluctuation data was primarily applied to suppress low‐frequency background variations, including fixed‐pattern noise, that could bias NPS estimation. The detrended fluctuation data were subsequently subjected to a 2D Fourier transformation. Finally, the one‐dimensional (1D) NPS was calculated using the radial frequency method,^27^ as also described in AAPM Report No. 233 for CT image noise analysis,^28^ which involves averaging values at each radial distance from the origin of the frequency domain (i.e., for each frequency).

### 2.3 Imaging system and software for noise reduction

Image acquisition was performed using an FPD CXDI‐720C (CANON MEDICAL SYSTEMS CORPORATION, Tochigi, Japan). The images had a pixel size of 0.125 mm × 0.125 mm, a matrix size of 2800 × 3408, and were stored in 16‐bit format. X‐ray exposure was performed using the X‐ray system DHF‐155HIII/UH‐6GE‐31E (Hitachi Medical Corporation, Tokyo, Japan), equipped with a total Al filtration of 2.8 mm. In this evaluation, image acquisition was performed without the use of either physical or virtual scatter reduction techniques.

Two noise reduction methods available in the console used with the CXDI‐720C imaging system were evaluated: a conventional rule‐based algorithm (conventional noise reduction, Con‐NR) and a DL‐based method (intelligent noise reduction, INR).^18^, ^19^, ^20^, ^21^, ^22^ According to available information,^18^, ^19^ the INR algorithm uses neural networks trained on carefully selected X‐ray images with varying noise levels and dose conditions, covering multiple and a wide range of anatomical sites; although the detailed architecture has not been disclosed by the manufacturer. Both methods allow adjustment across 10 levels of noise reduction intensity. For this study, levels 5 (moderate) and 10 (the strongest) were used for both methods. The proposed metric NPSIF(u) was applied to assess and compare operational characteristics, particularly the behavior of noise properties, when using uniform and phantom images, as detailed in the following sections.

All image data were exported in the DICOM format. Image handling and NPS calculation were performed using ImageJ (NIH, Bethesda, MD, USA). The radial frequency method for 1D NPS derivation, as well as additional data processing and visualization, such as numerical aggregation and plotting, were performed using Microsoft Excel (Microsoft Corporation, Redmond, WA, USA). No additional filtering, windowing, or nonstandard image processing steps for the analysis were applied beyond these procedures.

### 2.4 Evaluation of noise characteristics in uniform images

To evaluate noise characteristics under standard exposure conditions, uniform images were acquired using the RQA5 beam quality, as specified in IEC 61267.^29^ The X‐ray tube operated at 71 kV with a 21 mm‐thick Al filter mounted at the tube window to generate the RQA5 beam. The source‐to‐detector distance (SID) was set to 150 cm.

To assess the effect of radiation dose on image noise, the tube‐current‐time product was varied across three settings, 0.5, 1.0, and 5.0 mAs, while all other imaging parameters remained constant. In the uniform image experiments, a setting of 5.0 mAs was used to represent the standard dose condition, corresponding to a typical order of magnitude of incident X‐ray photons at the detector in routine imaging (10^5^ photons/mm^2^ scale), as estimated based on general clinical exposure conditions. An exposure of 1.0 mAs represented 20% of the standard dose and was considered the low‐dose condition, while 0.5 mAs represented 10% of the standard dose and was regarded as the ultra‐low‐dose condition, simulating an extreme dose reduction scenario. The acquired images were subsequently analyzed using ROIs placed in uniform areas at the center of the images to calculate noise metrics, including NPS and NPSIF, following the general ROI definition and NPS analysis procedure described in the previous section (B. Calculation of the noise characteristics). For uniform field images, noise analysis was performed directly on the acquired images, as no anatomical or structural patterns were present.

### 2.5 Evaluation of noise characteristics in phantom images

A chest phantom, PBU‐SS‐2 (Kyoto Kagaku, Kyoto, Japan) was used to evaluate the noise reduction effects on images containing anatomical structures. Measurements were performed at 70 kV with no additional filtration. The SID was set to 120 cm to simulate actual bedside clinical imaging conditions with the wireless FPD used in this study. To assess the effect of exposure level on noise reduction, the tube‐current‐time product was adjusted to settings of 0.5, 1.0, and 5.0 mAs. In the chest phantom experiments, a setting of 5.0 mAs was used to represent the standard dose condition, corresponding to an entrance surface dose of approximately 0.2 µGy to the patient's body. This dose level is comparable to the diagnostic reference level for bedside chest radiography and reflects typical clinical practice.^30^ A setting of 1.0 mAs, corresponding to 20% of the standard dose, was used as the low‐dose condition, while 0.5 mAs, corresponding to 10% of the standard dose, was used as the ultra‐low‐dose condition, representing a further substantial reduction in exposure.

Under each dose condition, three images were acquired with identical exposure settings. For phantom images, three independent pairs were formed, and three residual noise images were obtained by subtracting one image from another in each pair. This subtraction process isolates random noise by minimizing the effect of fixed anatomical structures, thereby enabling a more accurate evaluation of noise characteristics in structured regions.^31^ The NPS and NPSIF were calculated from each pair, and the final results were obtained by averaging across the three pairs. Because the subtraction process of two independent images increases the noise intensity by a factor of $\sqrt{2}$, the resulting NPS values were divided by 2 to correct for this effect.

**FIGURE 2:**
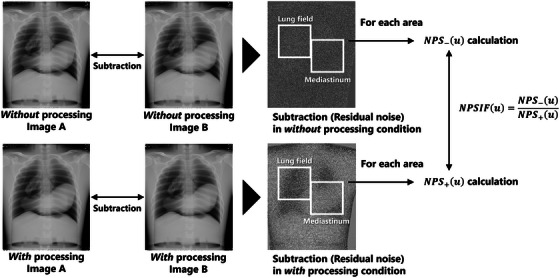
Calculation method of NPSIF(u) in phantom images. NPSIF(u) is calculated separately for the lung field and mediastinum ROIs. The size of each ROI is 1024 × 1024 pixels. The NPS calculation was performed using 256 × 256 pixel ROIs following the procedure shown in Figure 1.

In the phantom image analysis, the potential nonlinearity of noise‐reduction processing was assessed by considering the influence of the local anatomical background. Two representative regions in chest imaging, the lung field and mediastinum, were analyzed separately, and the corresponding ROI settings are shown in Figure 2.

## 3 RESULTS

### 3.1 Noise characteristics in uniform image conditions

**FIGURE 3:**
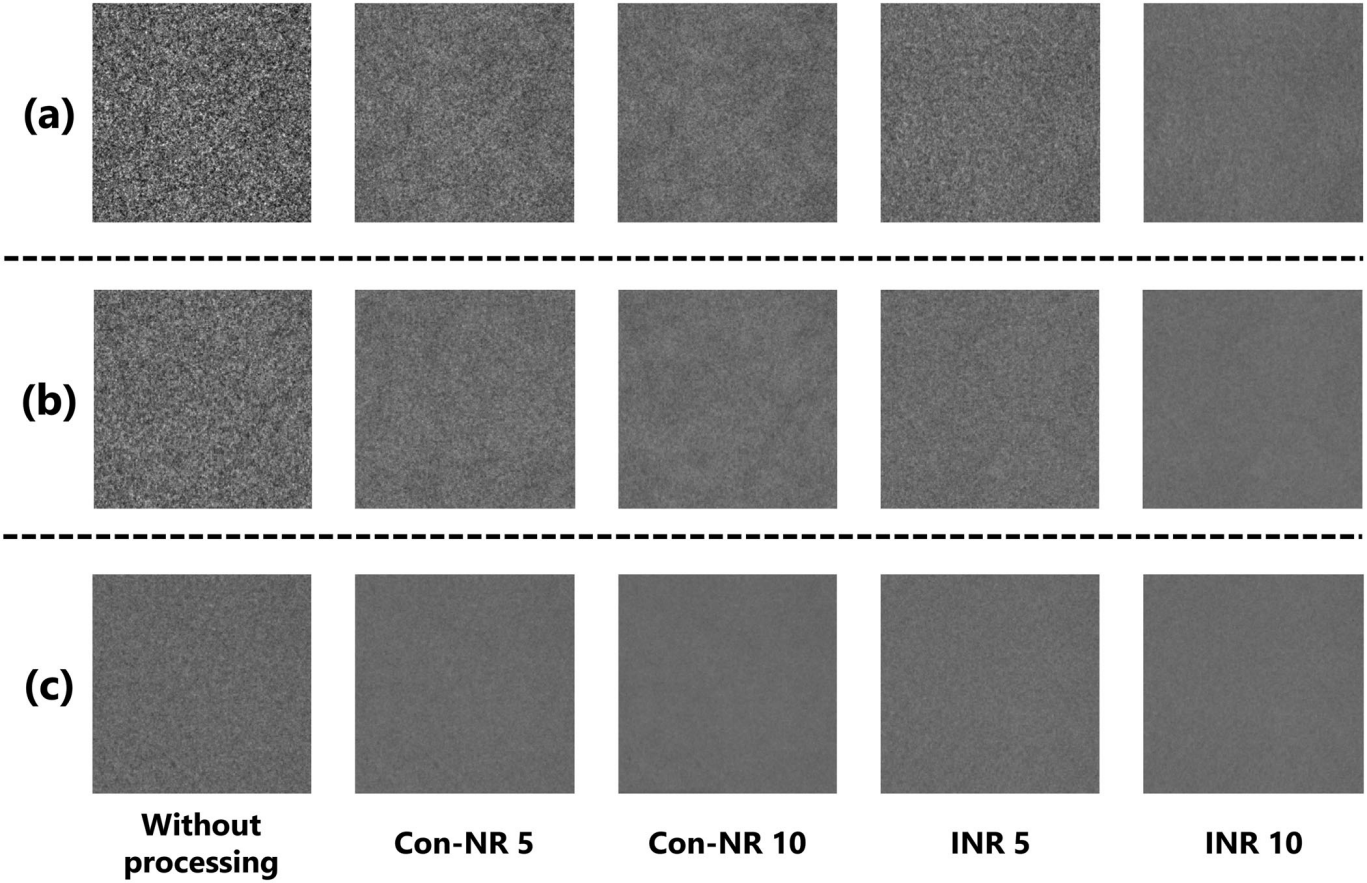
Differences in the appearance of uniform noise images under various processing conditions: (a) ultra‐low dose (0.5 mAs), (b) low dose (1.0 mAs), and (c) standard dose (5.0 mAs). The images were processed using either the INR or Con‐NR, where the numerical values (e.g., INR 5, Con‐NR 10) indicate the corresponding noise reduction intensity levels. The window level is the mean pixel value, and the window width is 100 for display. The 256 × 256 image is extracted from the center of the 1024 × 1024 image.

Figure 3 presents uniform images acquired at three dose levels: (a) ultra‐low dose (0.5 mAs), (b) low dose (1.0 mAs), and (c) standard dose (5.0 mAs). A visual assessment indicates that the effectiveness of noise reduction varies with the algorithm and dose level, with the most pronounced differences observed under ultra‐low‐dose conditions.

**FIGURE 4:**
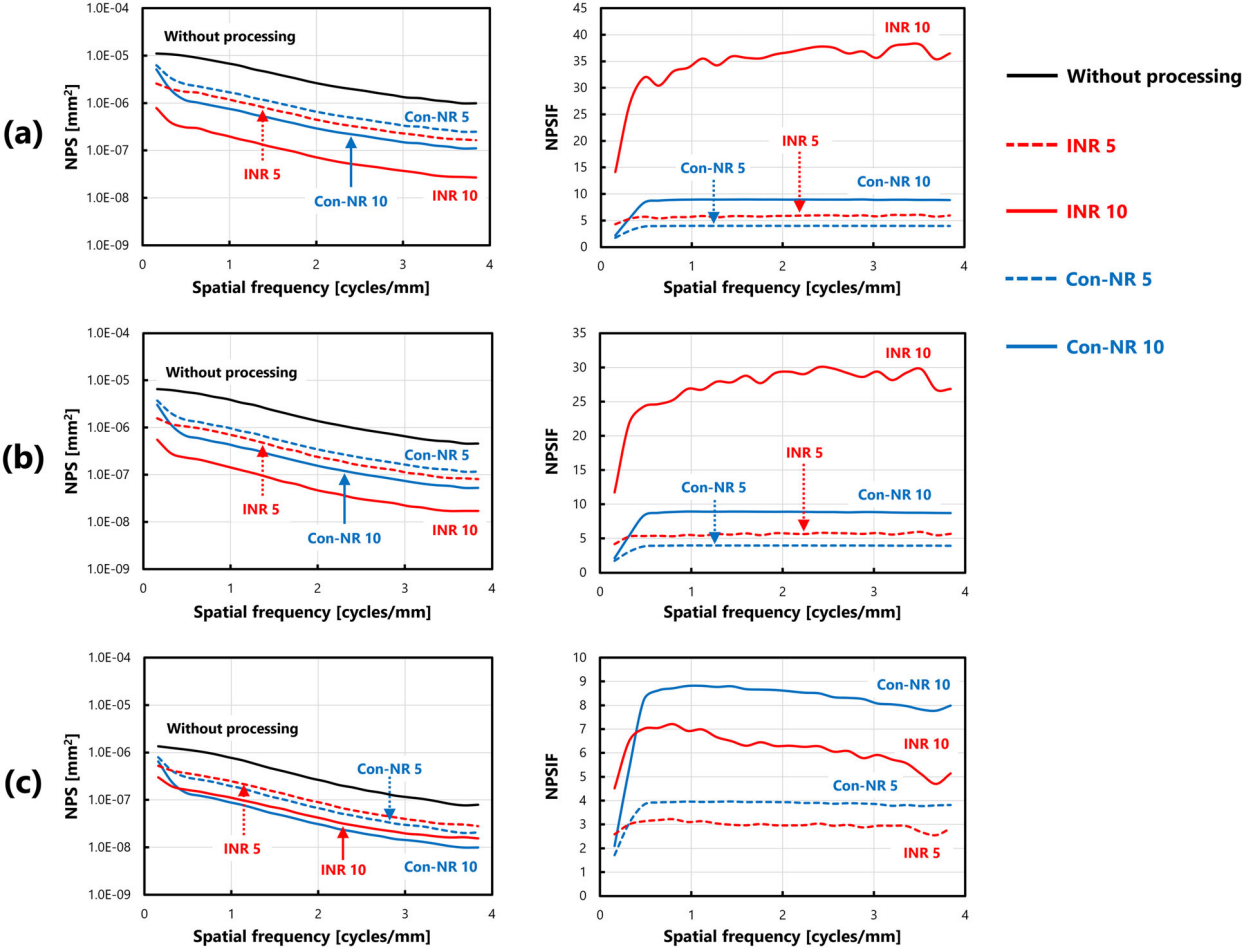
Comparison of NPS characteristics and calculation results of NPSIF(u) under various processing conditions: (a) ultra‐low dose (0.5 mAs), (b) low dose (1.0 mAs), and (c) standard dose (5.0 mAs).

Figure 4 shows the NPS and NPSIF for each dose condition: (a) ultra‐low dose (0.5 mAs), (b) low dose (1.0 mAs), and (c) standard dose (5.0 mAs). Under ultra‐low‐dose conditions, noise‐reduction processing reduced overall NPS values. Furthermore, evaluations based on the NPSIF revealed consistent noise reduction effects across all spatial frequencies, with particularly significant improvements under the INR 10 condition. Under low dose conditions, the trends in both the NPS and NPSIF were similar to those observed at the ultra‐low dose level, thereby confirming the effectiveness of the noise reduction techniques. In contrast, under standard dose conditions, the NPS distribution exhibited a different trend than at the lower dose levels. NPSIF assessment indicated that the effect of INR processing on noise reduction was less pronounced than that of the Con‐NR. Notably, under the INR 10 setting, the NPSIF values decreased with increasing spatial frequency, thereby suggesting a reduced effect at higher frequencies.

**FIGURE 5:**
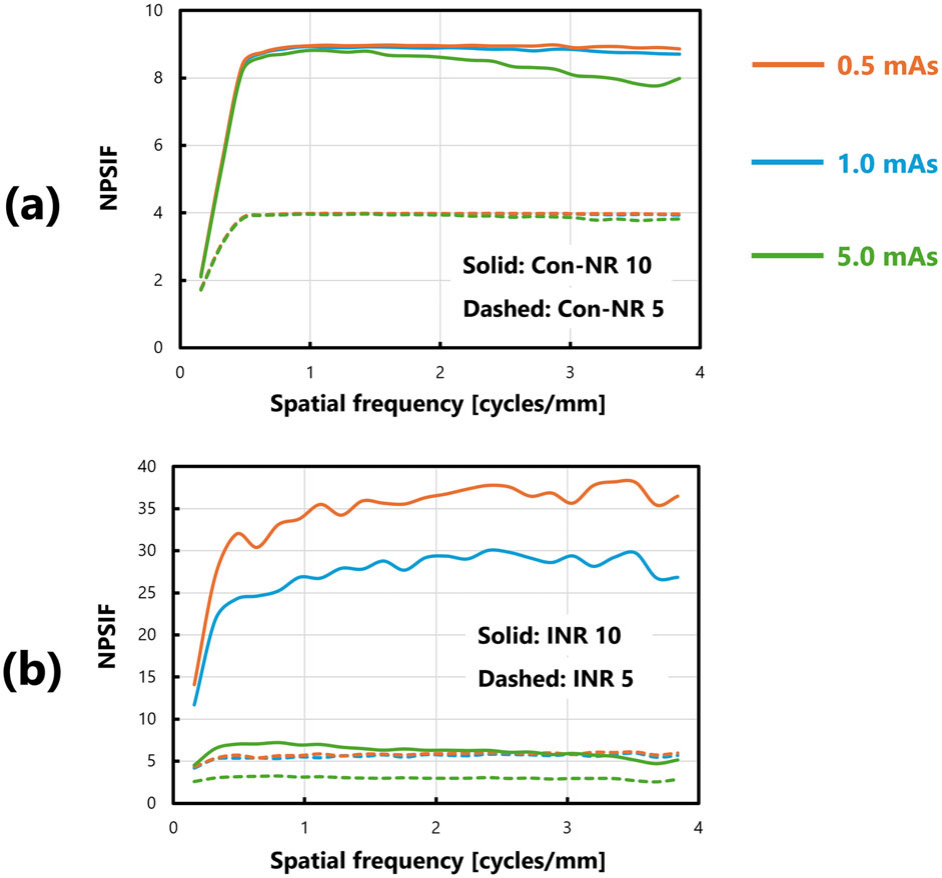
Comprehensive comparison of NPSIF(u) under all dose conditions: (a) Con‐NR model‐based processing, and (b) INR processing using DL. Solid lines indicate the results when the processing intensity is 10, whereas dashed lines indicate the results when the intensity is 5.

A comprehensive comparison of the NPSIF across all dose levels revealed that, although the effectiveness of INR processing varied with dose, Con‐NR demonstrated a consistent effect regardless of dose. Figure 5 presents a reorganized summary of the NPSIFs shown in Figure 4 for each processing method, facilitating clearer interpretation of their behavior. This figure does not represent newly calculated data, but rather a restructured presentation of the same results.

### 3.2 Noise characteristics in chest phantom image conditions

**FIGURE 6:**
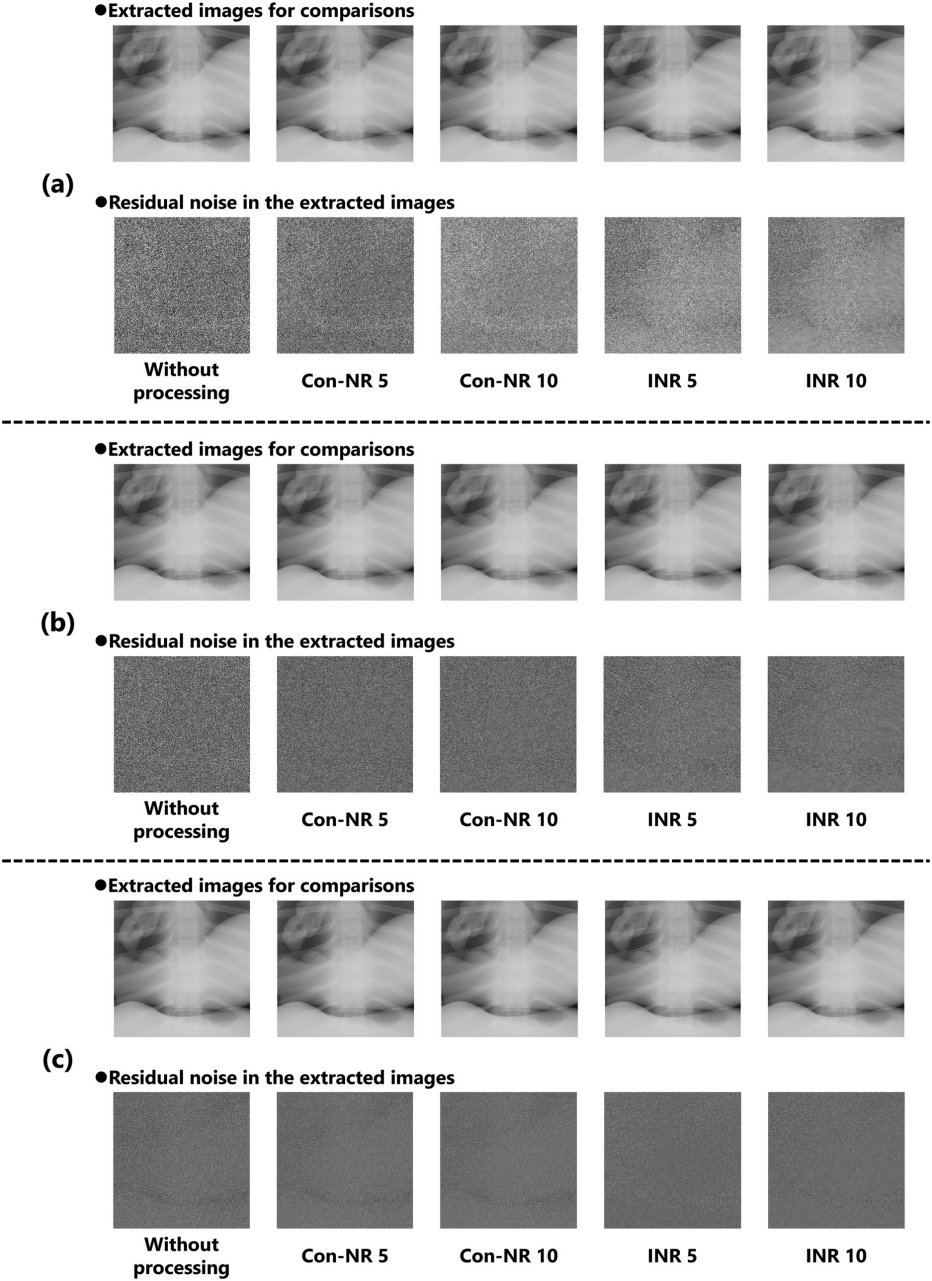
Differences in the appearance of phantom images under various processing conditions: (a) ultra‐low dose (0.5 mAs), (b) low dose (1.0 mAs), and (c) standard dose (5.0 mAs). For the phantom images, the window level was set to the mean pixel value and the window width to 3000. For the residual noise images, the window level was 0 and the window width was 300. Residual noise images were obtained via image subtraction.

Figure 6 illustrates the chest phantom images acquired at three levels: (a) ultra‐low dose (0.5 mAs), (b) low dose (1.0 mAs), and (c) standard dose (5.0 mAs). These images were extracted solely for qualitative visualization of noise reduction effects in both the lung fields and the mediastinum, rather than for ROI‐based noise analysis. Accordingly, the ROIs shown here are different from those used in the quantitative analyses described below. Visual differences in the residual noise texture can be observed across different dose settings, which reflect the varying effectiveness of the applied noise‐reduction techniques.

**FIGURE 7:**
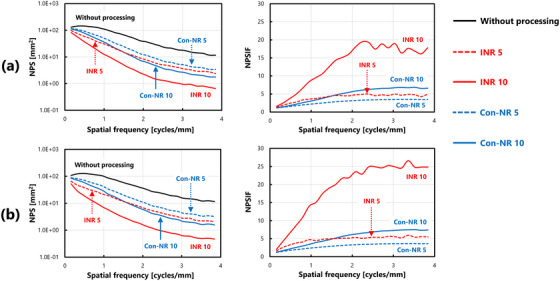
Comparison of NPS characteristics and calculation results of NPSIF(u) under various processing conditions: ultra‐low dose condition (0.5 mAs): (a) Lung field and (b) mediastinum.

**FIGURE 8:**
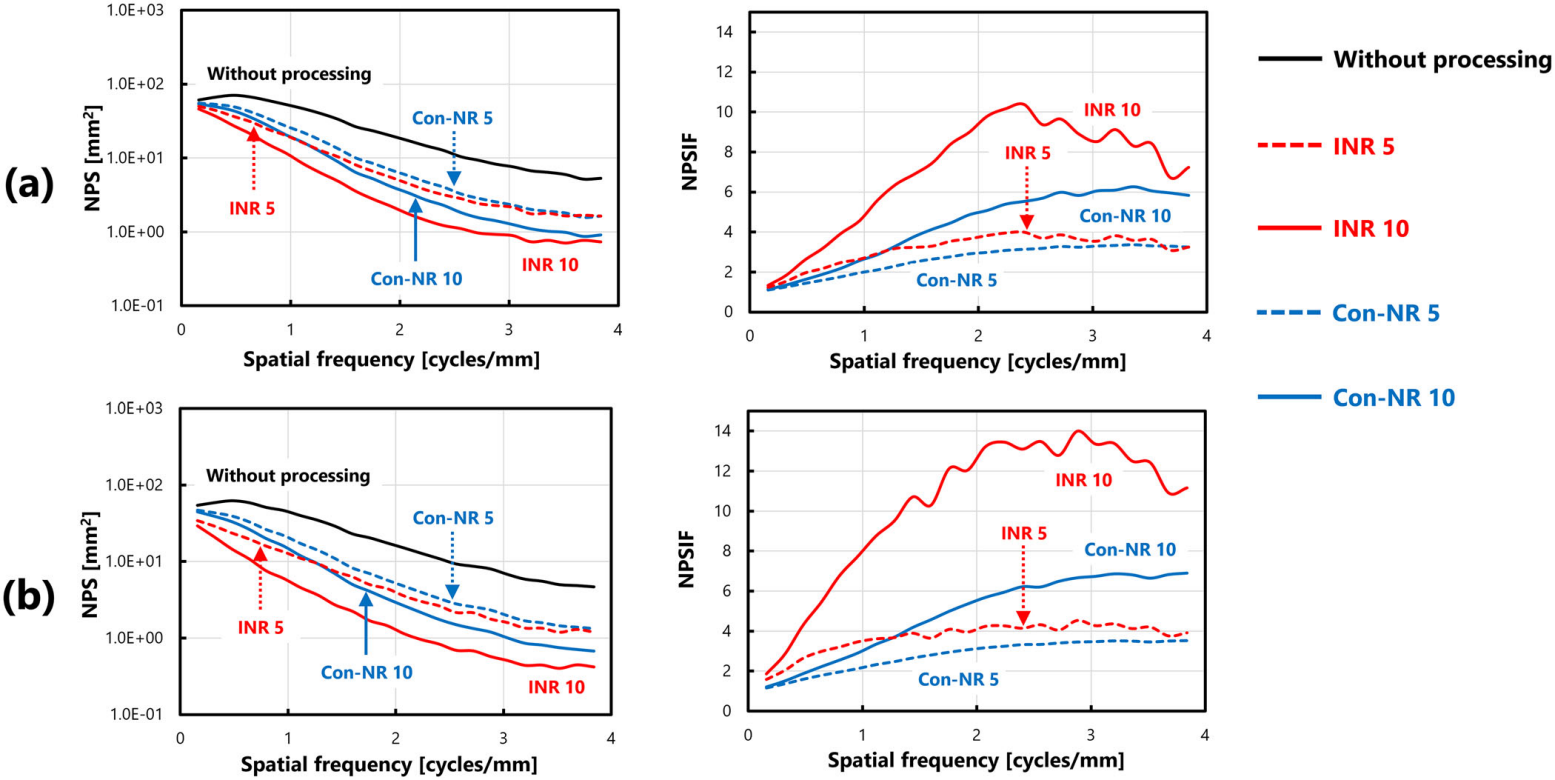
Comparison of NPS characteristics and calculation results of NPSIF(u) under various processing conditions: low‐dose condition (1.0 mAs): (a) Lung field and (b) mediastinum.

**FIGURE 9:**
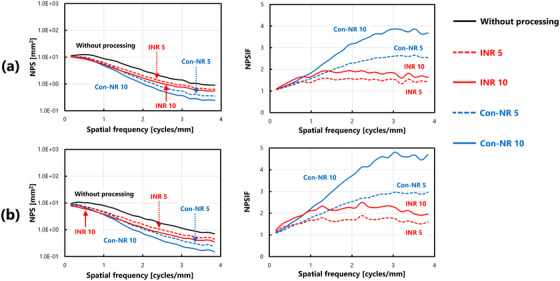
Comparison of NPS characteristics and calculation results of NPSIF(u) under various processing conditions: standard dose condition (5.0 mAs): (a) Lung field and (b) mediastinum.

Figure 7 shows the results obtained under ultra‐low dose conditions. Noise reduction processing reduced NPS values even in regions with complex anatomical structures, specifically in the lung field and mediastinum, which were investigated in this study. However, NPSIF analysis revealed a non‐uniform pattern along the spatial frequency axis, which differed from the consistent improvement observed in uniform images. Figure 8 shows the results under low‐dose conditions. The trend of the change in the NPS was similar to that observed under lower‐dose conditions; however, NPS values did not change significantly with INR processing. The NPSIF of INR processing indicated a more pronounced dependency on the spatial frequency, with clear variations across frequencies. Figure 9 shows the results obtained under standard dose conditions. Under these conditions, noise reduction processing resulted in minimal changes to the NPS values. The evaluation of the NPSIF revealed that although the improvement effect of Con‐NR processing was smaller, the frequency‐dependent changes followed a trend similar to that of the other dose conditions. In contrast, under INR processing, the NPSIF remained nearly constant across spatial frequencies, indicating minimal improvement in the NPS.

**FIGURE 10:**
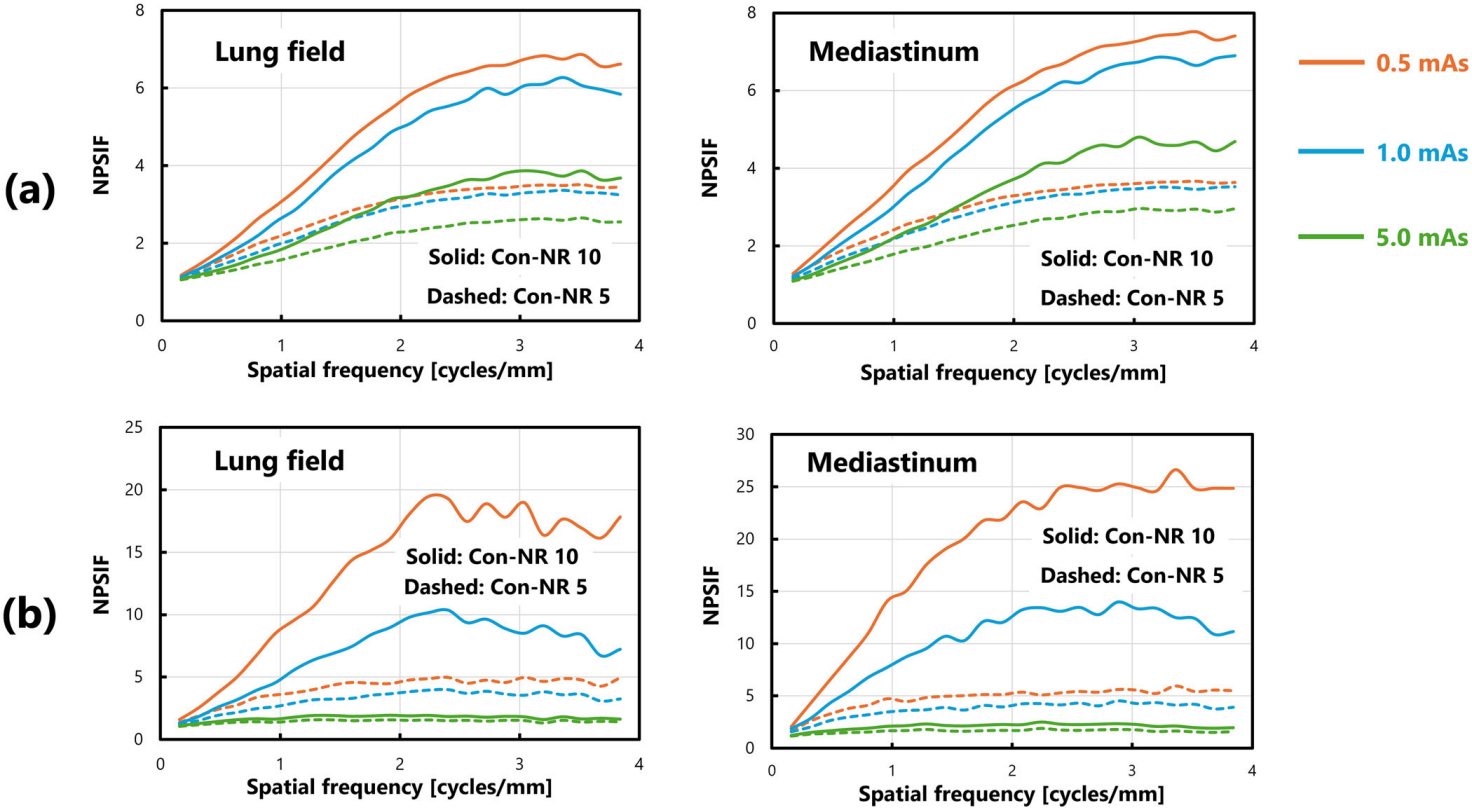
Comprehensive comparison of NPSIF(u) under all dose conditions: (a) Con‐NR model‐based processing, and (b) INR processing using DL. Solid lines represent results with a processing intensity of 10, whereas dashed lines represent results with an intensity of 5.

Under all dose conditions, Con‐NR processing exhibited a tendency for the NPSIF to increase with spatial frequency. On the other hand, INR processing improved up to a specific spatial frequency, beyond which it remained constant, thereby highlighting a distinct characteristic. Figure 10 provides a reorganized summary of the NPSIF results shown in Figures 7, 8, 9 to facilitate a more straightforward interpretation.

## 4 DISCUSSION

In this study, the NPSIF was used as a metric to characterize changes in image noise resulting from noise reduction processing. The NPSIF is conceptually related to the SNR improvement factor, SIF(u), which has been proposed as a comprehensive index to evaluate improvements in imaging performance by comparing SNRs before and after imaging processing such as utilizing anti‐scatter grids.^24^, ^25^ In contrast to SIF(u), which reflects the combined effects of resolution and noise, the NPSIF is defined solely as the ratio of the NPS before and after noise reduction processing. By isolating the noise component of the imaging chain, the NPSIF enables a direct assessment of how noise reduction algorithms modify the spectral distribution of image noise across spatial frequencies. This formulation allows the NPSIF to complement conventional image quality metrics by highlighting frequency‐dependent noise suppression characteristics that may not be evident in global noise measures. In particular, the NPSIF facilitates a detailed comparison of noise reduction behaviors across different processing techniques under identical acquisition conditions. Specifically, it played an essential role in comparing the improvement effects of conventional and DLNR processing, thereby aiding the understanding of frequency dependence and its limitations. The ability to visualize and quantify improvements at specific frequency ranges provides clinically meaningful insights, particularly when balancing noise suppression with the preservation of anatomical detail.

The results demonstrated that each noise reduction process, as evaluated by the NPSIF, exhibited distinct behavior across the spatial frequencies. The DL‐based INR algorithm demonstrated particularly substantial improvements in NPS under lower dose conditions, whereas its effect was diminished at higher doses. This trend has also been reported in previous studies on DL algorithms for medical image quality improvement,^32^, ^33^ supporting the validity of the present findings. The observed dose‐dependency in the NPSIF results suggests that the characteristics of its training data may constrain DLNR's performance. DL‐based denoising algorithms often learn to reproduce the statistical distribution of noise present in the training dataset.^34^, ^35^ When such an algorithm is applied to images whose noise characteristics lie outside the training domain, the algorithm's ability to further reduce noise is limited. Consequently, the upper limit of achievable noise improvement could be primarily influenced by the noise distribution included in the training data. In fact, a previous study highlighted that improvements across varying dose levels are not always consistent, suggesting limited generalizability beyond the training regime.^36^, ^37^ Furthermore, in domain‐adaptive noise reduction frameworks, several efforts have been made to address shifts in noise statistics across different dose levels, because simple DL models struggle when the noise characteristics (e.g., noise distribution or texture) in the test dataset falls outside the training distribution.^38^, ^39^, ^40^ Taken together, these considerations suggest that the performance of DLNR can be implicitly limited by the noise statistics of the training data when applied to images with different noise distributions. The dose‐dependent pattern observed in our study supports this interpretation and highlights the importance of recognizing the dose ranges within which clinical DL‐based image processing can operate robustly.

When applying new and distinct image processing techniques, it is essential to conduct comprehensive evaluations from multiple perspectives to fully understand their operational characteristics. By accurately identifying these characteristics, the benefits of such technologies can be more effectively translated to patient care. This study thoroughly evaluated the characteristics of a new noise reduction technique using NPS‐based analysis; however, some limitations should be considered when interpreting the findings. In this study, we focused on noise characteristics and analyzed the behavior of noise suppression using the NPSIF as an evaluation approach. While the NPSIF provides a frequency‐dependent description of processing‐induced changes in noise characteristics, it should be interpreted with appropriate caution. As a ratio‐based metric, the NPSIF can be sensitive to frequency‐dependent fluctuations in the underlying NPS estimates, particularly at spatial frequencies where the absolute noise power is low. In addition, the use of a ratio inherently removes information about the absolute magnitude of image noise, and the NPSIF does not directly reflect perceptual relevance or clinical impact. Therefore, the NPSIF would be better regarded as a complementary tool for characterizing relative noise suppression behavior across spatial frequencies, rather than as a standalone indicator of overall image quality or diagnostic performance.

In particular, a more comprehensive assessment that incorporated metrics related to diagnostic performance, such as spatial resolution and contrast, was beyond the scope of this study, which focused only on noise characteristics. Furthermore, to understand the clinical applicability of this technique, future research should explore integrated evaluation frameworks that combine NPS‐based analysis with other task‐specific metrics.^36^, ^41^, ^42^, ^43^, ^44^, ^45^ Additionally, this study did not evaluate the extent to which the radiation dose could be reduced by applying this technology, nor did it assess the potential impact on lesion detectability. Further studies are warranted to clarify the utility and clinical adaptability of new image processing techniques from a more comprehensive perspective.

## 5 CONCLUSION

In this study, we evaluated the image quality of a new DLNR algorithm (INR) for DR by comparing it with a conventional processing method. We introduced NPSIF, a novel metric that enabled a detailed and intuitive assessment of noise reduction performance across spatial frequencies. Through this approach, we demonstrated that the INR provided substantial noise improvements under lower dose conditions. In contrast, its effects were diminished at higher doses, revealing a dose‐dependent limitation likely related to the characteristics of its training data for DL technology. These findings highlight the usefulness of the NPSIF in distinguishing the operational characteristics of noise reduction processing techniques with different underlying mechanisms and support its role as a complementary tool to conventional noise metrics. Overall, the results of this study provide quantitative insights that may inform the selection and optimization of image‐processing methods, thereby supporting medical imaging teams in optimizing diagnostic image quality and patient radiation doses.

## AUTHOR CONTRIBUTIONS

All authors contributed to the study design and data interpretation. Experimental measurements were conducted by Hiroki Saito and Sho Maruyama. Sho Maruyama wrote the article and contributed to the entire study procedure as the principal investigator.

## CONFLICT OF INTEREST STATEMENT

The detector and mobile X‐ray device used in this study were loaned by CANON Medical Systems.

## ETHICS STATEMENT

This study was based entirely on physical phantom image or simulation data and did not involve human participants or patient data. Therefore, ethics approval was not applicable.

## Data Availability

The measured data that support the findings of this study are available from the corresponding author upon reasonable request.
